# The impact of an emotionally expressive writing intervention on eating pathology in female students

**DOI:** 10.1080/21642850.2018.1491797

**Published:** 2018-06-30

**Authors:** N. Kupeli, U. H. Schmidt, I. C. Campbell, J. Chilcot, C. J. Roberts, N. A. Troop

**Affiliations:** a Marie Curie Palliative Care Research Department, Division of Psychiatry, University College London, London, UK; b Section of Eating Disorders, Institute of Psychiatry, Psychology & Neuroscience (IoPPN), King’s College London, London, UK; c Health Psychology Section, Institute of Psychiatry, Psychology & Neuroscience (IoPPN), King’s College London, London, UK; d School of Health and Social Science, London South Bank University, London, UK; e Department of Psychology and Sport Sciences, University of Hertfordshire, UK

**Keywords:** Expressive writing, stress, BMI, disordered eating

## Abstract

**Introduction:** Previous research demonstrating emotional influences on eating and weight suggest that emotionally expressive writing may have a significant impact on reducing risk of eating pathology. This study examined the effects of writing about Intensely Positive Experiences on weight and disordered eating during a naturalistic stressor.

**Method:** Seventy-one female students completed an expressive or a control writing task before a period of exams. Both groups were compared on BMI (kg/m^2^) and the Eating Disorder Examination – Questionnaire (EDE-Q) before the writing task and at 8-week follow-up. A number of secondary analyses were also examined (to identify potential mediators) including measures of attachment, social rank, self-criticism and self-reassurance, stress and mood.

**Results:** There was a significant effect of intervention on changes in the subscales of the EDE-Q (*p* = .03). Specifically, expressive writers significantly reduced their dietary restraint while those in the control group did not. There was no significant effect of the intervention on changes in BMI or the other subscales of the EDE-Q (Eating, Weight and Shape Concern). There was also no effect of writing on any of the potential mediators in the secondary analyses.

**Discussion:** Emotionally expressive writing may reduce the risk of dietary restraint in women but these findings should be accepted with caution. It is a simple and light touch intervention that has the potential to be widely applied. However, it remains for future research to replicate these results and to identify the mechanisms of action.

## Introduction

Changes to eating and eating pathology can occur during periods of stress such as long working hours (e.g. Wardle, Steptoe, Oliver, & Lipsey, 2000) and in students sitting unseen exams (e.g. Roberts, Campbell, & Troop, 2014; Roberts, Troop, Connan, Treasure, & Campbell, 2007). Methods to reduce the impact of stress on eating behaviours therefore need to be identified. One promising method may be expressive writing (EW). To test this we evaluated the effect of EW on eating-related outcomes in female students over the course of taking unseen exams.

In this introduction, we first discuss a model of affect regulatory processes and how these relate to eating pathology. We then present evidence for EW and consider how EW may address these affect regulatory processes.

### Affect regulatory processes

Gilbert’s tri-partite affect regulatory model (Gilbert, 2005) is an interactive model consisting of the threat-defence system and two positive affect systems. The two positive affect systems capture resource/achievement-focused and affiliation-focused processes whilst the threat-focused component of this model refers to behaviours that are activated in response to threat and harm. Although the model emphasises reciprocal effects between these systems, the positive affect systems have been suggested to encourage the activation and immobilisation of the threat-defence system. The threat-defence system can be activated in response to perceived and actual danger. For example, when efforts to be valued by others are unsuccessful, this can result in a perception of low social rank which can activate the threat-defence system. However, a well-developed attachment system at times of threat can promote the ability to self-soothe by easily recalling memories of the safe and loving environment provided by a caregiver in childhood which can alleviate feelings of threat (Gilbert, 2005). Although both affect systems are related to psychopathology, it has been suggested that there is a difference between an activated resource-seeking system and a suppressed affiliation-focused system on adulthood psychopathology (Gilbert, Cheung, Grandfield, Campey, & Irons, 2003). The former refers to a focus on threats to social status suggesting that psychopathology ensues when an individual adopts a sense of low rank compared to those in its social environment whilst the latter indicates the inability to self-soothe at times of stress due to a lack of parental attentiveness during childhood (Gilbert et al., 2003). Therefore, the development of secure attachments is proposed to help us to learn the skills needed to manage difficult emotions at times of stress, being able to self-soothe rather than be self-critical (a form of self-attacking, associated with shame and a perceived low status; Gilbert, Baldwin, Irons, Baccus, & Palmer, 2006).

### Affect regulatory processes in eating behaviour and disordered eating

Affect regulatory processes have been shown to influence the experience of stress and are related to eating pathology. Insecurity of attachment (e.g. Cacioppo, Berntson, Sheridan, & McClintock, 2000), unfavourable social comparison (Dickerson & Kemeny, 2004; Kupeli et al., 2017; Stroud, Salovey, & Epel, 2002) and self-criticism (Gruen, Silva, Ehrlich, Schweitzer, & Friedhoff, 1997; Kupeli et al., 2017) have been associated with increased stress levels. Similarly, pathological eating behaviours have also been shown to be associated with unfavourable social comparison (e.g. Connan, Troop, Landau, Campbell, & Treasure, 2007; Troop, Andrews, Hiskey, & Treasure, 2014), vulnerable attachment (e.g. Zachrisson & Skårderud, 2010) and self-critical thoughts and feelings (Feinson & Meir, 2012).

### Expressive writing

Following Pennebaker and Beall’s (1986) seminal study on EW (which typically involves writing for 15–20 min for 3–5 consecutive days about a stressful or traumatic experience), findings have been somewhat equivocal. Predominantly, reviews and meta-analyses suggest that EW has a beneficial effect on a range of social, behavioural, psychological and health outcomes (Baikie & Wilhelm, 2005; Frattaroli, 2006; Frisina, Borod, & Lepore, 2004; Harris, 2006; Lowe, 2006; Meads, Lyons, & Carroll, 2003; Sloan & Marx, 2004a; Smyth, 1998; Wright & Chung, 2001), although some reviews suggest that EW does not produce a momentous positive effect on most physical and psychological outcomes (Meads & Nouwen, 2005; Mogk, Otte, Reinhold-Hurley, & Kröner-Herwig, 2006; Reinhold, Bürkner, & Holling, 2018). However, the reviews which have found an effect have reported small to medium effect sizes (Cohen, 1992), whilst Lowe (2006) refers to EW as being ‘mightier than the pill’ (p. 62).

Perhaps of most significance for the present study, EW within a therapeutic environment can aid recovery from eating disorders (East, Startup, Roberts, & Schmidt, 2010; Robinson & Serfaty, 2008). It has also been found to buffer the effects of stress on eating pathology in a student sample (Arigo & Smyth, 2012) and to improve perceptions of body image in a sample of undergraduate students (Lafont, 2011).

### Adaptations of expressive writing

Several studies have explored the effectiveness of EW by modifying the duration and/or frequency of writing (Burton & King, 2008; Chung & Pennebaker, 2008) and location (laboratory versus at home: van Middendorp, Sorbi, van Doornen, Bijlsma, & Geenen, 2007). It has also been shown to be effective across different age groups and professions as well as for people with different types of problem or condition (e.g. relationship difficulties, trauma, birth and psychological and physical illness) (Baikie & Wilhelm, 2005; Gordon, Baucom, & Snyder, 2004; Merz, Fox, & Malcarne, 2014; Pennebaker & Seagal, 1999; Sloan & Marx, 2004b). These findings illustrate the flexibility of EW and suggest that EW may be a cost-effective and easy to administer intervention for managing the effects of stress.

While most studies have asked participants to write about trauma, studies using positive writing tasks have found beneficial effects including improved health, a decreased risk of mortality, an increase in life satisfaction and a reduction in self-critical thoughts (Burton & King, 2004, 2008, 2009; Danner, Snowdon, & Friesen, 2001; King, 2001; King & Milner, 2000; Low, Stanton, & Danoff-Burg, 2006; Marlo & Wagner, 1999; Troop, Chilcot, Hutchings, & Varnaite, 2013; Wing, Schutte, & Byrne, 2006). Positive writing tasks include writing about previously experienced positive events (Burton & King, 2004; Marlo & Wagner, 1999), positive emotions in relation to illness (Low et al., 2006), perceived benefits of a traumatic experience (King & Milner, 2000) and writing about the future with a positive outlook (King, 2001; Troop et al., 2013). While these studies have shown a similar effect on health outcomes as trauma-writing, the advantage is that, in the short-term, there is an increase in positive mood (in contrast to the short-term increase in negative mood in trauma-writing studies).

### Theoretical underpinning of expressive writing

The approach adopted here is that of emotion regulation which suggests that it is the emotional arousal following expressive disclosure itself that is the important component rather than the stimulus that produced the response (Greenberg, Wortman, & Stone, 1996; Lepore, Greenberg, Bruno, & Smyth, 2002; Lowe, 2006; Quoidbach, Mikolajczak, & Gross, 2015). The development of mastery, self-efficacy and control over one’s emotions and the development of a self-soothing and accepting approach towards one’s thoughts and feelings is the key therapeutic process (Cameron & Jago, 2008; Greenberg et al., 1996; Lepore et al., 2002). In contrast, the information processing account (Pennebaker, 1997) suggests that the effect of EW is due to cognitive restructuring and the development of a new understanding of the trauma. However, this account cannot explain how the same health benefits can be obtained after writing about a trauma that participants had never experienced (Greenberg et al., 1996), although an emotion regulation account can. Therefore, EW may not reduce the perception of stress *per se*, but it attenuates the impact of stress (Lepore, 1997).

The emotion regulation interpretation of the processes driving EW can also be applied to the promising effects of positive forms of therapeutic writing. The function of positive expressive writing guides people through a process of structuring their thoughts and feelings to gain a better understanding of the emotions associated with the experience. For example, writing about the perceived benefits of a traumatic experience provide the writer with the opportunity to enhance their ability to deal with the emotions by allowing them to focus on the positive aspects of the event without the need to re-live the negative episode (King & Milner, 2000). Similarly, writing about intensely positive experiences (IPEs) or about a best possible self in the future provide an opportunity to enhance self-regulatory processes which not only induces positive affect but also reinforces the ability to arrive at a better understanding of one’s emotions and needs (Burton & King, 2004). In relation to Gilbert’s tri-partite model described earlier, studies on writing about IPEs have shown that the events participants describe tend to be either achievement-focused or relationship focused (Burton & King, 2004, 2008). These would be expected to stimulate the rank and attachment systems respectively and, subsequently, de-activate the threat (stress) system by reducing levels of self-criticism and increasing self-reassurance.

### Predicting change through text analysis

Studies using the Linguistic Inquiry and Word Count (LIWC; Pennebaker, Booth, & Francis, 2007) have revealed that improvements in health are associated with a greater use of positive emotion words with a moderate number of negative emotion words and an increasing use of cognitive words over the writing period (Pennebaker, 1997). In relation to eating behaviours/pathology, Chung (2009) found that successful dieters who use an online blogging community to track and share their dieting progress use more positive emotion words compared to those who are not as successful at achieving their weight loss goals. Users of pro-anorexia sites make fewer self-references but use more positive emotion and fewer cognitive mechanism words (Lyons, Mehl, & Pennebaker, 2006). Similarly, Wolf, Theis, and Kordy (2013) revealed that pro-ED bloggers wrote in a closed-minded fashion, were less emotionally expressive and featured more eating-related material compared to recovery bloggers. Text analysis of emails from ED patients to their therapists found a positive correlation between the number of words written and symptom improvement (Robinson & Serfaty, 2008). While there appears to be a link between the types/numbers of words used and their association with positive outcomes, we make no specific predictions about these in the present study. Nevertheless, a particular focus will be on the number of words used as well as the frequency of cognition and emotion words.

### The present study

Roberts et al. (2007, 2014) found an effect on weight and eating pathology in women who were experiencing a naturally occurring stressor (an unseen exam at the end of a taught module). This study, therefore, also took advantage of a naturally occurring stressor (students undertaking exams) and delivered an affect regulation intervention, writing about intensely positive experiences (IPEs) to *reduce* the impact of stress and measure its effect on eating pathology and weight.

The primary outcomes, therefore, are reductions in eating pathology and weight. Secondary outcomes are those relating to changes in stress, mood and affect regulation (since these are the processes by which EW has been proposed to work). The study will also use the text analysis (using the LIWC; Pennebaker et al., 2007) to identify psychological processes expressed in participants’ writing that may predict positive change.

### Research questions

In summary, research suggests that EW is an effective method of regulating emotions at times of difficulty and affect regulatory processes can influence the ability to regulate weight and eating pathology. Therefore based on previous research, the current study has been designed to resolve the following empirical questions:

1. Will writing about Intensely Positive Experiences (IPEs) influence changes in eating pathology and weight during an exam period?
2. Will changes in eating pathology and weight be due to changes in affect regulatory systems and processes?

## Methodology

### Participants

**Figure 1.**
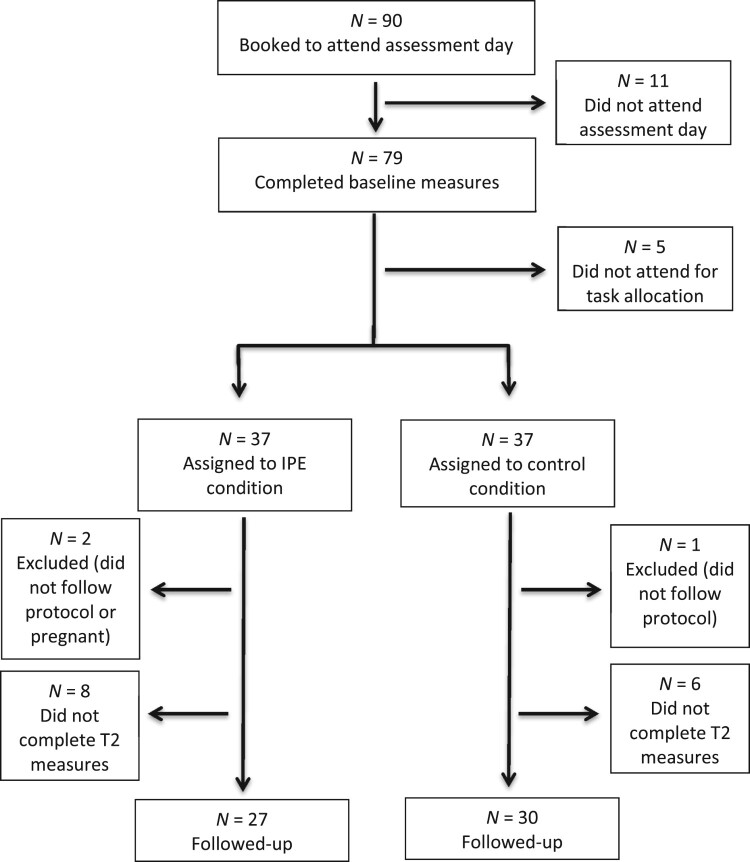
Flow chart for the number of participants who completed each phase of the study and attrition.

All female students from the undergraduate Psychology programme were eligible to participate. Of 90 individuals who volunteered to participate, 79 female students completed the T1 (baseline) measures of whom 74 returned to be allocated to a writing condition and 57 completed T2 (follow-up). Figure 1 provides details of participant retention and drop-out. Data from three participants were excluded (two IPE and one control); two did not follow study protocol and one became pregnant by time T2. After exclusions, 35 were in the IPE group and 36 in the control group, of whom complete data were available for 57 participants, 27 in the expressive writing group and 30 in the control group. Completers (*n* = 57) and non-completers (*n* = 14) did not differ significantly on any baseline variables (*p*-values between .11 and 1.00). Demographic information for participants allocated to the control and IPE groups for T1 of the study are presented in Table 1 which shows that the majority of the sample were British and single.

**Table 1.** Baseline demographic variables.

Variable	Total (*n* = 71)	IPE (*n* = 35)	Control (*n* = 36)	Significance
Age *M* (*SD*)	20.38 (4.04)	20.54 (5.18)	20.22 (2.55)	*t*(69) = −.33, *p* = .74
*Ethnicity *n* (%)*				
British	44 (62.0)	23 (32.4)	21 (29.6)	*χ*^2^ (df = 10) = 10.41 *p* = .41
Other European	3 (4.2)	2 (2.8)	1 (1.4)	
Indian	6 (8.5)	1 (1.4)	5 (7.0)	
Bangladeshi	1 (1.4)	1 (1.4)	0	
Pakistani	2 (2.8)	0	2 (2.8)	
Caribbean	2 (2.8)	1 (1.4)	1 (1.4)	
African	4 (5.6)	1 (1.4)	3 (4.2)	
Mixed ethnicity	2 (2.8)	1 (1.4)	1 (1.4)	
Chinese	3 (4.2)	2 (2.8)	1 (1.4)	
Other	4 (5.6)	3 (4.2)	1 (1.4)	
*Marital status *n* (%)*				
Single	40 (56.3)	15 (21.1)	25 (35.2)	*χ*^2^ (df = 5) = 7.96 *p* = .16
Married/Cohabiting	8 (11.3)	4 (5.6)	4 (5.6)	
In a relationship	21 (29.6)	14 (19.7)	7 (9.9)	
Divorced	1 (1.4)	1 (1.4)	0	
Widowed	1 (1.4)	1 (1.4)	0	
*Highest education *n* (%)*				
A Levels	67 (94.4)	34 (47.9)	33 (46.5)	Fishers exact test*: p* = .61
Bachelors	4 (5.6)	1 (1.4)	3 (4.2)	

### Measures and procedure

Recruitment of participants took place during undergraduate Psychology lectures at a University in the United Kingdom in which participants were invited to attend an assessment day. In addition to height and weight measurements, during this assessment participants also completed the following measures online using the Bristol Online Survey (BOS; University of Bristol, 2012) facility:

#### Primary outcome measures

The *Eating Disorder Examination Questionnaire* (EDE-Q; Fairburn & Beglin, 1994) was used to assess disordered eating. The EDE-Q is a 36-item questionnaire consisting of 22 items measuring disordered eating attitudes and behaviours and 14 diagnostic items. Items include ‘Have you tried to avoid eating food which you like in order to influence your shape or weight?’ and ‘Have you had a strong desire to lose weight?’ For the purpose of the current study, the 14 diagnostic items were not used. The remaining 22 items were used giving subscale scores of dietary restraint, eating concern, weight concern and shape concern and the internal reliabilities (*α*) for the EDE-Q subscales were .84, .84, .82 and .92, respectively. High scores indicated more eating pathology.

In addition, height and weight measurements were taken by the researcher in order to calculate BMI kg/m^2^ objectively.

#### Secondary outcome measures

The *Perceived Stress Scale-4* (PSS-4; Cohen & Williamson, 1988) is a 4-item measure used to assess perceptions of stress over the last month. Each item is rated on a 5-point scale. Items include ‘In the last month, how often have you felt that you were unable to control the important things in your life?’ and ‘In the last month, how often have you felt that things were going your way?’ Positive items were reversed and high scores indicated more stress (*α* = .82).

The *Short Depression-Happiness Scale* (SDHS; Joseph, Linley, Harwood, Lewis, & McCollam, 2004) was used to measure mood. The SDHS has 6 items with lower scores indicating greater depressed mood and higher scores indicating greater happiness (*α* = .89). Items include ‘I felt dissatisfied with my life’ and ‘I felt that life was meaningless’.

The *Social Comparison Rating Scale* (SCRS; Allan & Gilbert, 1995) was used to measure social rank. The SCRS is an 11-item measure, which requires respondents to rate how they judge themselves in comparison to others. The items state ‘In relation to others I feel … ’ and each item is rated on a 10-point Likert scale with dimensions such as inferior-superior, left out-accepted and unattractive-more attractive. High scores indicate a favourable comparison, low scores an unfavourable social comparison (*α* = .94).

*Vulnerable Attachment Style Questionnaire* (VASQ; Bifulco, Mahon, Kwon, Moran, & Jacobs, 2003) was used to measure behaviours, emotions and attitudes indicating insecurity of attachment. Items include ‘I rely on others to help me make decisions’ and ‘I find it hard to trust others’. Higher scores indicate a more vulnerable attachment. The present study used a briefer and psychometrically improved 14-item version (Kupeli et al., 2015) (*α* = .81).

*Forms of Self-Criticising/Attacking and Self-Reassuring Scale* (FSCRS; Gilbert, Clarke, Hempel, Miles, & Irons, 2004) was used to measure self-criticism/reassurance. Positive items reflect the ability to self-reassure (referred to as reassured-self [RS]) and negative items indicate self-critical thoughts and feelings (split into subscales of inadequate-self [IS] and hated-self [HS]). Items include ‘I am easily disappointed with myself’ and ‘I am gentle and supportive with myself’. The present study used a briefer and psychometrically improved 18-item version (Kupeli, Chilcot, Schmidt, Campbell, & Troop, 2013) (*α*’s for the RS, IS and HS scales were .90, .92 and .88, respectively).

### Procedure

Within two weeks of completing the baseline measures, participants returned to be assigned to the writing condition. A quasi-random allocation method was used to allocate alternate participants to writing conditions. Participants were provided with a notebook and asked either to write about an IPE or about a control topic for 15 minutes each day for 3 consecutive days. All participants were advised to complete the writing task at home and return it to the researcher within 6 weeks.

The following instructions were given to participants in the IPE condition (adapted from Burton & King, 2004):

Think of the most wonderful experience in your life, happiest moments, ecstatic moments, moments of rapture, perhaps from being in love, or from listening to music, or suddenly ‘being hit’ by a book or painting or from some great creative moment. Choose one such experience or moment. Try to imagine yourself at that moment, including all the feelings and emotions associated with the experience. Now write about the experience in as much detail as possible trying to include the feelings, thoughts, and emotions that were present at the time. Please try your best to re-experience the emotions involved. All of your writing will be completely confidential. Don’t worry about spelling, sentence structure, or grammar. The only rule is that once you begin writing, continue to do so until your time is up.

Participants in the control writing condition were instructed to write a review of a film or book they had recently seen or read whilst remaining objective (as adapted from Troop et al., 2013). On the second and third days of writing, participants were instructed to either write about the same positive experience/film or book as the day before or they could choose to write about a different positive experience/film or book.

Following each diary entry, participants were also required to complete a 3-item scale measuring how personal and meaningful they felt their diary entry was and to rate their mood. These items were measured using a 7-point Likert scale ranging from −3 to + 3 with high scores indicating that the diary entry was more meaningful and personal and that they felt happy immediately post-writing. The IPE diaries were typed up and the texts were analysed using the LIWC (Pennebaker et al., 2007).

Participants were advised that they would be contacted via text message to inform them of when they would need to complete the follow-up online self-report measures (which took place approximately 8 weeks after baseline and 2 weeks before their exams). Shortly after their exams had finished, participants attended a final weigh-in and collected their £15 Amazon voucher. All participants were provided with a debriefing sheet and an information leaflet about specialist services set up to assist individuals who suffer from mental health problems such as depression or EDs.

### Statistical analysis

A series of independent samples *t*-tests compared the immediate effects of writing (averaged across the 3 days of writing) in IPE versus control participants on ratings of mood and how personal and meaningful participants felt their writing was.

Intention to treat and analyses as per protocol were conducted to examine the longer term effects of writing about IPEs. For BMI, SDHS, SCRS, PSS-4 and VASQ, repeated measures ANOVA with one between-subjects (*condition*: control versus IPE) and one within-subjects (*time*) variable were conducted. As the EDE-Q and FSCRS have subscales, repeated measures ANOVA with one between-subjects (*condition*: control versus IPE) and two within-subjects (*time* and *subscale*) variables were computed. For any significant effects found, pairwise comparisons using the Bonferroni adjustment were computed to determine where these differences were present.

Finally, the LIWC (Pennebaker et al., 2007) programme was used to assess the expressive writers’ language use and the changes in the types of words used over the course of the writing task. The entries were assessed for general measures such as word count, linguistic dimensions such as prepositions and pronouns and psychological processes such as positive and negative emotion words, cognition words (such as those indicating causal reasoning and insight) and social words (such as references to family and friends). Contingent on the results of the repeated measures ANOVAs conducted to assess the enduring effects of writing about IPEs on various outcome measures, where a significant improvement is found, language use was compared between those who improved and those who did not. In order to assess this, univariate ANOVAs were conducted comparing improvers and non-improvers on the various LIWC measures.

Finally, where appropriate, effect sizes were examined using partial eta-squared (*η*²) with values of .01, .06 and .16 representing small, medium and large effect sizes, respectively (Cohen, 1977).

### Ethics statement

The study received ethical approval from the University of Hertfordshire Ethics Committee (reference: PSY/09/11/NK).

## Results

### Sample characteristics

Means and standard deviations are presented in Table 2. All variables were normally distributed and there was no significant difference between groups at baseline. Mean scores at baseline are of a similar magnitude to those of a recent large community-based sample of women (Kupeli et al., 2017).

**Table 2.** Results of the repeated measures ANOVA’s based on intent to treat analysis comparing control and IPE groups on mean (*SD*) pre- and post-intervention scores for variables of interest.

Variable	Control (*n* = 36)		IPE (n = 35)		Interaction	
	T1	T2	T1	T2	Effects	*η*^2^
*Primary outcomes*						
BMI	24.50 (4.70)	24.42 (4.69)	24.62 (6.17)	24.46 (6.12)	*F*(1, 69) = .09, *p* = .76	.001
*EDE-Q subscales*						
Dietary restraint	1.16 (1.57)	1.14 (1.61)	1.47 (1.28)	.87 (1.01)	*F*(3, 67) = 3.21, *p* = .03	.13
Eating concerns	1.16 (1.38)	1.24 (1.50)	1.00 (1.04)	.91 (.89)		
Weight concerns	1.87 (1.65)	1.94 (1.67)	2.03 (1.56)	2.03 (1.76)		
Shape concerns	2.35 (1.78)	2.22 (1.82)	2.49 (1.56)	2.53 (1.85)		
*Secondary outcomes*						
PSS-4	11.47 (3.61)	11.47 (4.16)	11.11 (2.71)	11.83 (3.29)	*F*(1, 69) = 1.57, *p* = .21	.02
SDHS	18.58 (4.12)	18.00 (4.24)	18.86 (3.86)	19.20 (3.71)	*F*(1, 69) = 3.01, *p* = .09	.04
SCRS	61.42 (18.21)	61.75 (17.71)	57.34 (17.13)	59.89 (18.28)	*F*(1, 69) = 1.05, *p* = .31	.02
VASQ (Total score)	38.61 (8.87)	40.39 (9.20)	37.80 (8.01)	39.37 (8.24)	*F*(1, 69) = .04, *p* = .85	.001
*FSCRS scales*						
Reassured-self	27.53 (7.20)	27.17 (7.20)	27.11 (6.15)	27.23 (6.09)	*F*(2, 68) = .42, *p* = .66	.01
Inadequate-self	16.81 (6.63)	16.86 (6.85)	18.40 (7.09)	17.83 (6.87)		
Hated-self	6.58 (3.82)	7.08 (4.31)	6.14 (3.65)	6.46 (3.62)		

### Immediate effects of writing

Ratings of mood and how personal and meaningful participants felt their diary entries to be were averaged across the three days. Compared with the control group, participants writing about IPEs reported significantly higher mood levels (*M *= 1.62 [*SD *= .76] vs *M *= .63 [*SD *= .93]; *t*_55_ = −4.38, *p* < .001) and indicated that they felt that their diary entries were more personal (*M *= 5.09 [*SD *= 1.19] vs *M *= 2.94 [*SD *= 1.15]; *t*_55_ = −6.89, *p* < .001) and meaningful (*M *= 5.51 [*SD *= .94] vs *M *= 2.91 [*SD *= 1.14]; *t*_55_ = −9.31, *p* < .001) following each writing session.

### Longer term effects of writing

Table 2 gives the results of the repeated measures ANOVA using intention to treat analysis. In terms of the primary outcomes, there was a significant 3-way interaction effect on the EDE-Q with medium to large effect sizes (*p* = .03, *n*^2^ = .13). Post-hoc analysis using a Bonferroni adjustment revealed that dietary restraint, but not the other EDE-Q subscales, significantly reduced in the IPE condition (*p* < .01). There were no significant reductions on any subscale in the control condition. There was no significant effect of the intervention on changes in BMI.

There was no significant Condition × Time effect on PSS-4, SCRS, VASQ or FSCRS scales. There was, however, a marginally significant effect of the intervention on mood with those who completed the IPE task reporting an increase in their mood (indicating more happiness) compared with those in the control group. These results did not differ when analyses was conducted using data collected from participants who completed all phases of the study (see Appendix 1 for study results as per protocol).

### Content and text analysis of writing about IPEs

The diary entries of participants writing about IPEs were coded to determine the types of themes that were disclosed. Themes included going on holiday (25.9%), interpersonal events (18.5%), finishing exams or receiving exam results for entry to University (11.1%), celebrations such as birthdays and Christmas (9.9%), attending a music concert or festival (7.4%), spending time with family and friends (6.2%), birth of a child including own child or sibling (4.9%), listening to a favourite song or watching a favourite film (3.7%), and other events such as getting married, starting a first job and taking part in a sky dive (12.4%).

As writing about IPEs was found to have a positive impact on dietary restraint levels, these participants were grouped into those who improved their restraint (*n* = 18) and non-improvers (*n* = 9). Analysis using the LIWC showed that improvers wrote significantly more words over the writing period compared to the non-improvers (means [*SD’s*] were 336.5 [147.4] versus 270.3 [65.9], *F*_1,79 _= 5.83, *p* = .02, *η*^2^ = .07) and used marginally fewer present tense words (means [*SD’s*] were 4.4% [2.8] versus 5.7% [3.7], *F*_1,79_ = 3.36, *p* = .07, *η*^2^ = .04). There were no other significant differences.

## Discussion

The present study examined the effect of writing about intensely positive experiences (IPEs) on eating behaviour and disordered eating in a group of female students undertaking exams. Furthermore, the degree to which positive outcomes could be attributed to changes in stress, affect systems (social rank and insecurity of attachment) and affect processes (self-criticism and self-reassurance) was also explored.

Writing about IPEs led to a significant reduction in dietary restraint and promoted marginal improvements in mood. These findings are in line with previous research in relation to disturbed eating behaviours (East et al., 2010) and mood (Baikie & Wilhelm, 2005; Pennebaker, 2004; Pennebaker & Seagal, 1999; Smyth, 1998). No significant effects were found for the other primary outcomes or, indeed, for any of the secondary outcomes. In other words, the changes in dietary restraint cannot be accounted for by changes in affect systems of social rank and attachment or by changes in affect regulatory processes of self-reassurance or self-criticism.

Within the IPE group, text analysis revealed that writing more words over the writing period is related to an improvement in restrictive behaviours. These results support findings by Robinson and Serfaty (2008) who found that engaging with and making the most of an EW task is related to an improvement in ED symptoms. However, no other psychological processes reflected in the text analysis showed any significant association with improvement.

### Strengths and limitations

The current study has a number of strengths, for example, the design included using a naturally occurring stressor. Students undertaking exams provided the optimal resource for exploring the effects of EW on stress-related changes in weight, eating behaviours and eating pathology. Secondly, although several of the constructs were assessed using self-report measures, BMI was measured objectively by the first author. However, we note that neither the IPE nor the control group reported an increase in stress in the lead up to exams and so it may be that the exam period was not experienced as stressful. This may be due to the recruitment of predominantly first and second year students for whom exams may not appear as important as they do to final year students.

The current study also has a number of limitations that must be acknowledged. The use of self-report questionnaires is one. However, as already noted, some measures were more objective. Another limitation of the current study is the final sample size which was reduced due to an attrition rate of 23% between pre- and post-intervention. Therefore, future studies should recruit a larger sample of women. Additionally, the present study did not employ a clinical sample of ED patients so the findings relating to restrictive eating behaviours may not be generalisable to a clinical population.

Finally, although the current study has high ecological validity as participants completed the writing task at home, it is also possible that participants misinterpreted or ignored writing task instructions. Having said that, studies have shown that disclosure can be successfully self-administered and home-based writing sessions generally produce larger effects (Frattaroli, 2006; van Middendorp et al., 2007). Furthermore, examination of participants’ writing suggested that all wrote about the topic to which they were assigned and the mood/personal/meaningful ratings after each writing episode also suggested participants were meaningfully engaged. We would also argue that for any self-directed intervention to be of value, it needs to be demonstrated to be effective when entirely self-administered rather than under the scrutiny of psychologists in an experimental lab.

### Implications

While an affect regulation task led to changes in dietary restraint, it is not certain that this was due to changes in affect regulation, either in terms of systems (social rank and attachment) or processes (self-criticism and self-reassurance). This could be because the measures used in this study are not sensitive to the change caused by EW. Alternatively, it could be due to the type of EW task that was used. Troop et al. (2013) found that writing about life goals resulted in a reduction in self-criticism (using the same measure of self-criticism/reassurance used in the present study). Perhaps writing about life goals may be more effective than writing about IPEs in influencing this change. It is not immediately clear why it was specifically dietary restraint that reduced and not other subscales of the EDE-Q. One could speculate that dietary restraint is a more threat-based construct, based on fear of weight gain and the risk of breaking dietary rules, while the other subscales include elements of dissatisfaction and the desire to change (improve) rather than just the fear of negative change. Future research should delineate the differential impact of EW on these related but different constructs. On the other hand, since the number of words used in writing about IPEs predicted who improved in dietary restraint and who didn’t, one focus of attention may be to ensure adequate levels of engagement and effort by participants. This may be particularly important when EW is being carried out at home rather than in a laboratory under the watchful eye of an experimenter.

Three meta-analyses concluded that disclosure in the form of EW does not produce positive health effects on most physical and psychological outcomes (Meads & Nouwen, 2005; Mogk et al., 2006; Reinhold et al., 2018). It may not be surprising, therefore, that writing about IPEs did not produce a change on most of the measures used in the present study (or indeed, in Burton and King’s (2004) original study). However, it is also possible that writing about IPEs produced changes in variables that were not measured here such as health centre visits, immune function and exam grade results (as previously shown in relation to trauma-writing: Pennebaker, 1997, 2004; Smyth, 1998).

Furthermore, because the focus of interest was on eating pathology, only women were recruited. However, it has been suggested that men show greater benefits from EW than women (Smyth, 1998).

No significant changes were identified in those affect regulatory variables relating to Gilbert’s (2005) model, namely social rank, attachment, self-criticism and self-reassurance. This study was not a test of Gilbert’s model per se, rather it adopted Gilbert’s model to provide a framework to understand the effect of writing about intensely positive experiences. It may be that the measures used were unable to capture the relevant processes adequately. For example, a measure of positive affect derived from Gilbert’s model may have identified changes in activated positive affect and safe/content positive affect (Gilbert et al., 2008) better than the associated achievement- and affiliation-related measures included here. Perhaps more likely still is that the writing task did not stimulate the relevant processes sufficiently well. For example, Gilbert’s (2005, 2009) model goes on to describe the role of self-compassion. Expressive writing studies asking participants to write from a self-compassionate perspective (e.g. Imrie & Troop, 2012; Leary, Tate, Adams, Batts Allen, & Hancock, 2007) may provide a better approach to stimulate these process.

Finally, it is possible that the lack of benefits from writing in this study is due to the fact that, with the recent development of virtual social networking, people may already disclose this kind of information on a daily basis. Therefore, as suggested by Smyth and Pennebaker (2008), the question is whether a time has come when we can discuss our emotions freely without the fear of stigma or judgment rather than inhibiting our responses. If so, the traditional writing paradigm may no longer be as effective as it was in a culture where expressing thoughts and feelings about stress and emotions was not a common occurrence. However, as no longitudinal research has explored time trends in the effects of the EW paradigm, currently this point is rather speculative. Therefore, this is a question that should be explored by future research or by meta-analysis looking at year of publication as a factor contributing to the beneficial effects of EW.

Although the current study did not employ a clinical sample, the findings still have implications for practice. Dietary restraint often leads to a greater likelihood of overeating at times of stress, so to find that writing about IPEs reduces dietary restraint may have important preventive implications. These findings also support previous recommendations that expressive disclosure within a therapeutic environment could be an effective adjunct to psychological and medical therapy for treating pathological eating patterns (Robinson & Serfaty, 2008; Schmidt et al., 2002) and as a self-help tool rather than as a therapeutic method on its own (Baikie & Wilhelm, 2005; Lowe, 2006; Marlo & Wagner, 1999; Wright & Chung, 2001).

## Conclusion

The current study presents evidence that writing about intensely positive experiences reduces dietary restraint. However, the mechanisms by which such writing leads to improvements require further investigation.

## Appendix 1

**Table A1.** Results of the repeated measures ANOVA’s as per protocol comparing control and experimental groups on mean (*SD*) pre- and post-intervention scores for variables of interest.

Variable	Control (*n* = 30)		IPE (*n* = 27)		Interaction	
	T1	T2	T1	T2	Effects	*η*^2^
*Primary outcomes*						
BMI	24.21 (4.19)	24.12 (4.16)	24.04 (6.33)	23.84 (6.37)	*F*(1, 55) = .11, *p* = .74	.002
*EDE-Q subscales*						
Dietary restraint	1.05 (1.55)	1.04 (1.60)	1.50 (1.30)	.73 (.89)	*F*(3, 53) = 3.41, *p* = .02	.34
Eating concerns	1.07 (1.27)	1.17 (1.43)	.97 (1.01)	.86 (.81)		
Weight concerns	1.61 (1.55)	1.70 (1.60)	2.08 (1.54)	2.09 (1.79)		
Shape concerns	2.22 (1.65)	2.05 (1.68)	2.42 (1.44)	2.47 (1.85)		
*Secondary outcomes*						
PSS-4	11.47 (3.30)	11.47 (4.01)	11.37 (2.57)	12.30 (3.24)	*F*(1, 55) = 1.17, *p* = .20	.03
SDHS	18.67 (3.85)	17.97 (4.01)	18.78 (3.92)	19.22 (3.72)	*F*(1, 55) = 2.97, *p* = .09	.05
SCRS	59.17 (16.71)	59.57 (16.10)	56.15 (17.19)	59.44 (18.82)	*F*(1, 55) = 1.16, *p* = .29	.02
VASQ (Total score)	38.33 (7.74)	40.47 (8.22)	38.22 (8.48)	40.26 (8.60)	*F*(1, 55) = .01, *p* = .94	<.001
*FSCRS scales*						
Reassured-self	27.03 (7.18)	26.60 (7.15)	26.78 (6.82)	26.93 (6.76)	*F*(2, 54) = .44, *p* = .65	.02
Inadequate-self	16.57 (6.48)	16.63 (6.75)	17.89 (7.03)	17.15 (6.67)		
Hated-self	6.20 (3.02)	6.80 (3.79)	5.81 (2.77)	6.22 (2.76)		
